# Everybody's Page

**Published:** 1891-09-26

**Authors:** 


### Everybody's Page.

So little provision exists in Spain for the treatment of women suffering from diseases peculiar to their sex, that a few months ago the Spanish Gynæcological Society appointed a committee to consider the advisability of establishing a Gynæcological Institute in Madrid. The committee has now reported that the creation of such an institution is urgently needed in each of the provinces as well as in the capital.

---

The accuracy of statistics, from which somewhat sweeping deductions are often made, is seldom beyond suspicion. This seems to have been exemplified in a remarkable manner in New York, where the City Board of Health is reported to have notified the physicians that there was neglect in reporting births. The result of this notification is alleged to be an increase in births in two succeeding weeks of from 697 to 1,288.

The practice of medicine and pharmacy combined is probably not so common in this country now as was the case many years ago. Of its being detrimental to the public as well as to the medical profession there can hardly be any doubt. In another respect we appear to have retrograded. Originally an apprentice to an apothecary served five years, and presumably gained in that time a fair knowledge of his subject. Now the Apothecaries' Hall only requires a three months' course of pharmacy and dispensary, and as there is no examination in practical dispensing, the work is generally done, as Mr. W. Martindale has asserted, in a very perfunctory manner. That it is so done is not a matter for surprise, but the public must be pardoned if sceptical of the accuracy with which their prescriptions are dispensed, and if they call for a reform in the training of the modern apothecary. In few things more than drugs is it true that "a little knowledge is a dangerous thing."

